# Apoptotic Resistance of Metastatic Tumor Cells in Triple Negative Breast Cancer: Roles of Death Receptor-5

**DOI:** 10.31557/APJCP.2019.20.6.1743

**Published:** 2019

**Authors:** Mohammad Kamalabadi-Farahani, Mohammad Reza H Najafabadi, Zahra Jabbarpour

**Affiliations:** 1 *Department of Tissue Engineering and Applied Cell Sciences, Faculty of Advanced Technologies in Medicine, *; 2 *Department of Medical Nanotechnology, School of Advanced Technologies in Medicine, *; 3 *Department of Molecular Medicine, Faculty of Advanced Technologies in Medicine Tehran University of Medical Sciences, Tehran, Iran. *

**Keywords:** Triple-negative breast cancer, metastasis, apoptosis, DR-5, curcumin

## Abstract

**Background:**

Metastasis is a major cause of death from cancer in triple-negative breast cancer (TNBC). Apoptosis evasion is a critical feature of metastatic tumor cells. Chemopreventive and apoptotic potential of curcumin has been shown in breast cancer. However, the precise mechanism of these effects against metastatic tumor cells has not been clearly addressed yet.

**Methods:**

4T1 cell line was used for induction of metastatic animal model of breast cancer. Primary and metastatic tumor cells were extracted from subcutaneous tumor and lung of cancerous mice, respectively. MTT assay was used to determine the effect of curcumin on viability of tumor cells. Quantitative real-time polymerase chain reaction was performed to analyze the effect of curcumin on death receptor-5 (DR-5) gene expression.

**Results:**

Our data revealed that, compared with primary tumor cells, metastatic tumor cells were more resistance to apoptosis effects of curcumin. The DR-5 gene expression was up-regulated in both primary and metastatic tumor cells after curcumin treatment, but this up-regulation was significantly higher in primary tumor cells compared with metastatic cells.

**Conclusion:**

These findings provided important insights regarding the molecular mechanism of apoptosis resistance of metastatic tumor cells and can be used for designing a targeted therapeutic strategies in combat with metastatic TNBC.

## Introduction

Breast cancer is the most common cancer among women worldwide. Triple-negative breast cancer (TNBC) is the most aggressive and invasive type of breast cancer with poor prognosis. Anthracyclines-based combination chemotherapy is the standard treatment for patients with TNBC (Yao et al., 2017). However, the recurrence and metastasis of TNBC due to chemoresistance takes place in up to 70% of the patients (Isakoff, 2010). Metastases account for 90% of human cancer deaths. In cancer, metastasis and resistance to chemotherapy are associated with each other (Acharyya et al., 2012). In breast cancer, metastasis is a major cause of death from cancer. Bone, lung, and liver are the main sites of metastases in this type of cancer (Gonzalez-Angulo et al., 2007). Chemoresistance often hampers tumor cells from undergoing sufficient levels of apoptosis, resulting in cancer cell survival and treatment failure (Wilson et al., 2009).

The apoptosis or programmed cell death is considered as an important homeostatic mechanism that equilibrates cell generation with cell death and maintains correct cell numbers in the body in physiological and pathological conditions (Martin and Green, 1995). Two fundamentally distinct apoptotic signaling pathways have been determined in mammalian cells, namely extrinsic (or death receptor pathway) and intrinsic (or mitochondrial) pathways (Igney and Krammer, 2002). The extrinsic pathways involve death receptors (DRs). The death receptors are members of the tumor necrosis factor receptor superfamily and include a subfamily that is characterized by an intracellular death domain. Among all the DRs, DR-4 and DR-5 are selectively expressed in tumor cells. Accordingly, these receptors provide specific option for targeted cancer therapy (Igney and Krammer, 2002; Srivastava, 2001).

It is documented that tumor growth is a result of both uncontrolled proliferation and reduced apoptosis. Therefor, inducing cancer cell apoptosis has been one of the key strategies in anticancer therapy (Tamm et al., 2001). Currently, there are several strategies for targeting apoptosis in breast cancer immunotherapy and chemotherapy. Besides TRAIL and FasL, apoptosis can be induced by various stimuli and through diverse mechanisms. However, development of resistance toward apoptosis is one major clinical challenge (Igney and Krammer, 2002; Stepczynska et al., 2001). 

Curcumin is a bright yellow colored polyphenol extracted from the rhizome of the plant *Curcuma longa L. (Zingiberaceae)*. The antiproliferative property of curcumin has been shown in vitro and in vivo against human breast cancer cells due to induction of apoptosis (Kumar et al., 2016) . The antitumor activity of curcumin has been demonstrated in a mouse model of breast cancer, demonstrating that curcumin supplemented diet inhibited tumor growth and angiogenesis (Bimonte et al., 2015). Curcumin can also significantly reduce the number of mice with lung metastasis in immunodeficient mice, in whom MDAMB231 cells were injected via intra-cardiac route (Bachmeier et al., 2007). Synergistic effect of curcumin and other anticancer drugs have been demonstrated in many research. In a recent study, synergistic effect of paclitaxel in combination with curcumin against human MCF-7 and MDAMB231 cells was demonstrated (Quispe-Soto and Calaf, 2016). Improved clinical responses were also observed in clinical trial of docetaxel plus curcumin in patients with advanced and metastatic breast cancer, informing curcumin as an appropriate agent in combination with other anticancer drugs (Bayet-Robert et al., 2010). 

Metastasis is a complex succession of cell-biological events in which certain tumor cells detach from the primary tumor, circulate in the vascular or lymphatic system, and finally exit from the blood or lymph and constitute a new tumor in appropriate tissue. Tumor cells in different steps of metastasis process have distinctive and appropriative properties that will help them during this process (Valastyan and Weinberg, 2011).

 Metastatic cells are cells originated from primary tumors. However, functional and structural futures of these cells differ from their parents. Metastatic cells tend to exhibit a greater survival ability and apoptosis resistance. It is postulated that the increase in apoptosis resistance of metastatic cancer cells is associated with modifying expression levels or function of proteins involved in apoptosis signaling pathways. However, there is little direct experimental evidence linking apoptosis and metastasis (Cameron et al., 2000). 

Apoptotic effects of curcumin have been proven in various cancers and tumor cells. However, the magnitude and mechanism of these effects in metastatic cells compared to their primary resources have not been clearly addressed yet. Therefore, in this study, we explored the viability of primary and metastatic tumor cells in response to curcumin as an apoptosis inducer. In addition, we compared the relative expression of the DR-5, as an important factor of apoptosis pathway, in primary and metastatic tumor cells following curcumin treatment.

## Materials and Methods

*Cell culture*

4T1 cell line was purchased from the cell bank of Pasteur Institute of Iran (C604). The cells were cultured in high glucose Dulbecco’s Modified Eagle’s Medium (DMEM) containing 10% FBS (fetal bovine serum) and 2% Penicillin-Streptomycin (all from Gibco, USA) in humidified atmosphere of 5% CO_2_ at 37°C. 

*Induction of syngeneic animal model of breast cancer*

Female BALB/c mice weighing 20 to 25 gram obtained from Royan institute (Iran). The animals were housed in cages at 12-h photoperiod while they had free access to food and water. All animal experiments were in compliance with the relevant laws, and this study was approved by the Ethics Committee of Tehran University of Medical Sciences (registration number: IR.TUMS.REC.1394.1439). 4T1 cells were subcutaneously injected to the flank (or the right hind limb) of the mice (10^5 ^cells suspended in 100 μL PBS) using an insulin syringe with 32G needle. The mice were monitored daily for the appearance and behavior characteristics. 

*Lung metastatic and primary breast tumor cell extraction*

For primary and metastatic tumor cell extraction, primary tumor and lung of cancerous mice were excised after 35 days of tumor induction in mice, and surface blood was removed by rinsing it in PBS. After mincing with scissors, fragments were placed to 50 ml conical tube. For enzymatic digestion, primary tumor and the lungs were digested in 10 mg ⁄ ml collagenase type IV at 37°C for 75 min on a platform rocker. All enzymes were purchased from Sigma (St Louis, MO, USA). The digested organ filtered through 70-um cell strainers, and washed with PBS. In the next step, washed cells were resuspended in medium containing 10% FBS, 100 U/ml Penicillin, and 100 ug/ml Streptomycin (all from Gibco, USA). Ultimately, the cells were cultured at 37°C in 5% CO_2_.

*MTT Assay*

The MTT tetrazolium assay was used to measure cell viability. Briefly, 1 × 10^3^ 4T1 cells were seeded in each well of 96-well plates in DMEM containing 10% FBS and 2% Penicillin-Streptomycin (all from Gibco, USA) in humidified atmosphere of 5% CO_2_ at 37°C. After 24 h incubation, cell culture medium was exchanged with complete medium supplemented with different concentrations (30 uM, 50 uM, 60 uM, 75 uM, 100 uM, 120 uM, and 125 uM) of curcumin. Following 48 h incubation at 37°C, medium was removed and 50 ml of MTT solution at 5 mg/ml (Sigma) was added to the cultures and the incubation continued for a further 4-h period, after which 150 ml of DMSO was added. Formed formazon crystals were allowed to dissolve for 30 min before measuring the optical density at 570 nm using mQuant plate reader (Bio-Tek Instrument, USA). Finally, cell viability was expressed as percent compared to control wells according to the following equation:

$\mathrm{Cell viability}=\frac{\left(\mathrm{absorbance of treated well}-\mathrm{absorbance of blank wells}\right)}{\left(\mathrm{absorbance of control well}-\mathrm{absorbance of blank wells}\right)}\times 100$

In this equation blank means culture medium without cells and control means culture medium with cells. This experiment was performed in triplicate.

*Quantification of DR-5 by RT-qPCR*

Briefly, primary and metastatic cells (1×10^4^) were seeded in each well of 24-well plates in complete medium. After 24 h incubation, the cells medium was exchanged with complete medium supplemented with 120 uM of curcumin. Following 48 h incubation, total RNA was extracted from these cells using QIAzol Lysis Reagent-QIAGEN. The quality, yield, and size of extracted RNA were analyzed using spectrophotometry (NanoDrop-ThermoFisher) and electrophoresis. The first strand cDNA synthesis was performed using reverse transcription system (PrimeScript™ RT reagent Kit (Perfect Real Time -Takara). Real-time PCR procedure was executed based on the 1 ng/μl cDNA in all samples. Quantization of all gene transcripts was done by real-time quantitative PCR using Power SYBR Green PCR Master Mix, and an RT-qPCR analysis for DR-5 was carried out by SYBR Green Real time PCR Master Mix (Amplicon A/S, Denmark) according to the respective manufacturer’s instruction. The amplification procedure was as follows: 1 cycle of 95°C for 15 min, 40 cycles of 95°C for 30 sec, 60°C for 30 sec, and 72°C for 30 sec. The exact mRNA expression was normalized to the expression level of GAPDH. Relative changes of gene expression were calculated by the following formula, and the data was represented as fold up-regulation/down-regulation.

Fold change = 2^-ΔΔCt^, where ΔΔCt = [Ct of DR-5 (in treated cells) - Ct of GAPDH (in treated cells)] - [Ct of DR-5 (in control cells) - Ct of GAPDH (in control cells)].

Primers were designed using AlleleID version 6 software. The used primers are as follows: for DR-5, Forward 5′-AAAACGGCTTGGGCATCTTGGC-3′, Reverse 5′-AGACGGTTCCAGGAGTCAAAGG-3′; for GADPH, Forward 5′-GGTGAAGGTCG GTGTGAACG-3′, Reverse 5′-CTCGCTCCTGGAAGATGGTG -3′.

RNA and DNA gel electrophoresis assay

Total extracted RNA from treated cells and PCR products were analyzed electrophoretically on 1.2% agarose gel containing 0.5 ug/ml safe stain(EB) (Sigma-Aldrich, MO, USA) for 1 h. DNA and RNA were visualized and photographed under transmitted ultraviolet light.

*Statistical analysis*

Results are expressed as the mean ± standard deviation. Data were analyzed with GraphPad Prism statistical software 6.0 (GraphPad Software, La Jolla, CA, USA) using Paired Samples t Test and two-way Anova. P <0.05 was considered statistically significant.

## Results

*Primary and metastatic tumor cells extraction*

**Figure 1:**
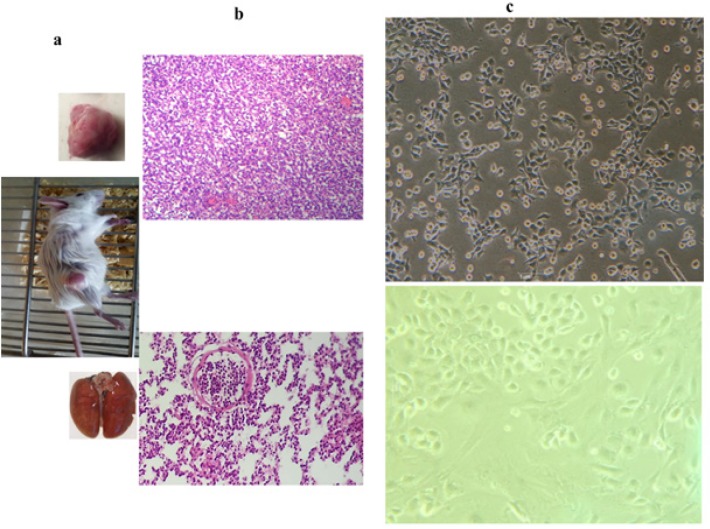
Primary and Lung Metastatic Tumor Cells Extraction. a. Metastatic animal model of triple negative breast cancer was generated after 35 days of tumor induction in Balb/c mice. b. H&E staining and pathological confirmation was performed on tumor tissues. c. Primary and metastatic tumor cells were extracted from subcutaneous tumor and lung of cancerous mice, respectively

Metastatic animal model of triple negative breast cancer was generated after 35 days following tumor induction in Balb/c mice (Figure 1a). When injected into BALB/c mice, 4T1 spontaneously produces highly metastatic tumors that can metastasize to the lung while the primary tumor is growing in situ. The primary tumor does not have to be removed to induce metastatic growth. 4T1 H and E staining and pathological confirmation were performed on tumor tissues (Figure 1b). We properly extracted primary and lung metastatic tumor cells from subcutaneous primary tumor and lung of cancerous mice, respectively. The metastatic tumor cells in the lung, after primary isolation, form colonies in the culture medium. Due to the high rate of growth and proliferation, the tumor cells in these colonies are purified after several passages. These metastatic tumor cells are called 4T1L while tumor cells that are obtained in the same way, from the original tissue of the tumor, are called 4T1P (Figure 1c).

*Compared with primary tumor cells, metastatic tumor cells are more resistance to apoptotic effects of Curcumin*

**Figure 2:**
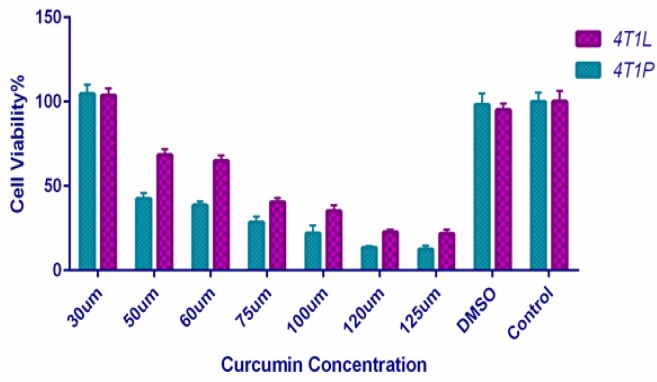
Tumor Cells Viability under Different Concentration of Curcumin. MTT assay results quantifying the viability of 4T1 cells under different treatment conditions. Untreated cells were used as the control. All results are expressed as mean ± SD. *P<0.05, **P<0.01

To determine the growth inhibi¬tory activity of curcumin on primary and metastatic tumor cells, 4T1P and 4T1L were treated with different concentrations of curcum¬in for 48 h, and viable cells were measured by MTT assay. The initial results indicated that curcumin-induced apoptosis were better analyzable qualitatively and statistically after 48 hours . Therefore, the MTT assay was only done in dose-dependent manner. Exposing 4T1P and 4T1L cells to curcumin result¬ed in a significant decrease in viable cells in a dose-dependent manner (Figure 2). Results indicated that metastatic cells were more resistance to apoptotic effects of curcumin at all concentrations. Effective concentrations were selected for all further mechanistic studies.

**Figure 3:**
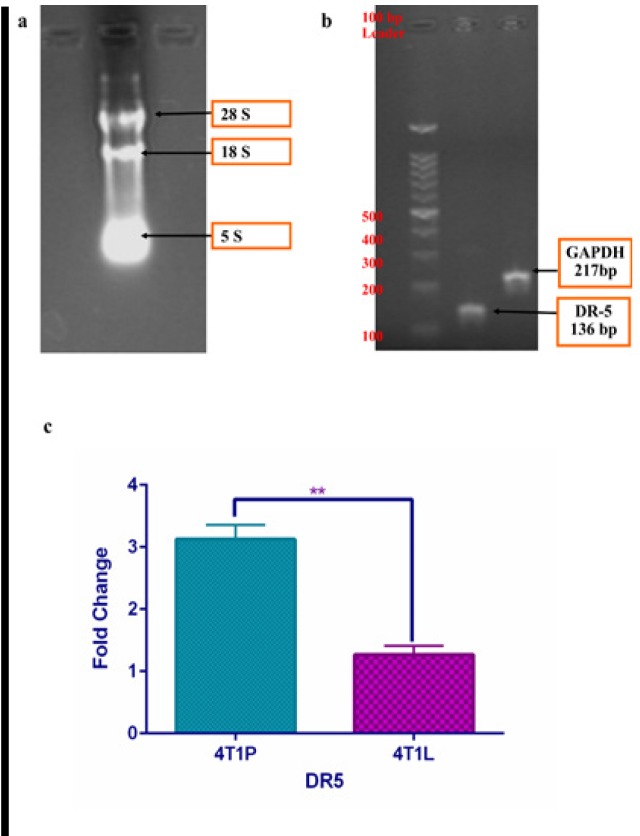
Enhanced Expression of DR-5 in 4T1L and 4T1P Cells after 48 h Treatment with Curcumin Using Real-Time PCR. a, b. The quality and size of extracted RNA and PCR products were confirmed using gel electrophoresis. c. Up-regulation of DR-5 in response to curcumin treatment was significantly higher in primary tumor cells compared with metastatic cells. All results are expressed as mean ± SD from at least three independent experiments analyzed by Two-tailed T test. **P < 0.001

*Role of DR-5 in susceptibility of 4T1L and 4T1P to apoptotic effects of Curcumin*

The process of programmed cell death or apoptosis is related to the activation of regulatory genes. Therefore, the expression level of its related genes was measured in this study. The expression of death receptor 5 (DR-5), considered as main receptors of TRAIL, was analyzed after curcumin treatment in 4T1L and 4T1P. Total RNA extraction, cDNA synthesis, and real time qPCR were done as described in material and method section . The quality, yield, and size of extracted RNA, synthesized cDNA, and PCR products were confirmed using nanodrope and gel electrophoresis (Figure 3a, b). After curcumin treatment, the expression of DR-5 was significantly up-regulated in both 4T1P and 4T1L. Curcumin treatment up-regulated DR-5 expression 3.1 times in 4T1P, but curcumin treatment up-regulated DR-5 expression 1.2 times in 4T1L (Figure 3c).Curcumin effects on DR-5 upregulation was significantly higher in 4T1P compared with 4T1L (Figure 3c). These results suggested that curcumin-induced cell growth inhibition and induction of apoptotic cell death involve the activation of DR-mediated pathway. We found that metastatic tumor cells were more resistant to curcumin-induced apoptosis. We also observed that DR-5 was involved in resistance of metastatic cells to apoptosis. 

## Discussion

Curcumin is a natural polyphenol with promising anticancer properties. Cancer cell-specific apoptotic effects of curcumin have been documented in many cancer cells (Kumar et al., 2016). In the present study, the effects of curcumin on metastatic tumor cells and their ancestors, primary tumor cells, were reviewed for the first time. Results of the present study demonstrated that curcumin inhibited the growth of primary and metastatic tumor cells. Our results clearly demonstrated that metastatic tumor cells were more resistance to the apoptotic effects of curcumin. 

Curcumin-mediated modulation of proliferation and apoptosis of breast tumor cells have been shown in many research (Wang et al., 2016). However, simultaneous assay of these effects in metastatic tumor cells and their ancestors, primary tumor cells, have not been shown yet. Results of a recent study in 2016 showed that curcumin analogues exhibited potent selective cytotoxic activity towards various human breast cancer-derived cells compared with non-malignant breast-derived cells. The aforementioned study revealed no significant differences in cytotoxic potencies of these analogues were observed between non-metastatic MDA-MB-231 cells and its metastatic variant MDA-MB-231/LM2-4 (Robles-Escajeda et al., 2016). In contrast to these results, we showed that metastatic cells were more resistance to the apoptotic effects of curcumin.

Breast cancer is the most common cancer in women worldwide, and comprises 18% of all cancers in women. Recent studies have focused on a greater understanding of the response and resistance to treatment, including the role of apoptosis (Prasad et al., 2014). In 2006, the TNBC was firstly described as a subtype of breast cancer with a more aggressive behavior and poorer prognosis. The aggressive clinical behavior and lack of molecular target conducted a lot of research on TNBC. In this regard, most of research has focused on chemo-resistance and metastasis as two main causes of relapse and eventually death of TNBC patients (Haffty et al., 2006). 

Decreased sensitivity to apoptosis induction plays critical roles in tumorigenesis, invasion, metastasis, and chemoresistance of breast cancers. Many questions regarding apoptosis resistance in breast cancer remain unanswered (L, 2006). Apoptosis, a form of programmed cell death, requires the caspases activation cascades. Procaspases activation is conducted from two major pathways, mitochondria-dependent and mitochondria-independent. Mitochondria independent pathway is associated with death receptors. Among the six different death receptors (DRs) identified to date, DR-4 and DR-5 are selectively expressed on cancer cells. Therefore, unlike chemotherapeutic agents, these receptors can mediate killing of tumor cells selectively (Prasad et al., 2014). 

Investigations showed that the cell growth inhibition by curcumin was performed through an apoptosis-dependent mechanism (Karunagaran et al., 2005). To elucidate the signaling pathways activated during curcumin treatment, we evaluated the expression of DR-5 as a key receptor protein known to participate in the apoptotic pathways. In the present study, we showed that growth-inhibition effects of curcumin and curcumin-induced apoptosis of tumor cells were associated with DR-5-mediated cell death pathway. We also indicated that curcumin induced an increase in the expression of DR-5 in primary and metastatic tumor cells. Analysis of DR-5 expression elucidated that curcumin-mediated upregulation of the DR-5 was significantly higher in the primary tumor cells than in the metastatic cells. Jung et al., reported that curcumin significantly induced DR-5 expression both at the mRNA and protein levels, accompanying the generation of the reactive oxygen species (Jung et al., 2005). In another study, it was demonstrated that curcumin enhanced TRAIL-induced apoptosis through induction of DR-5 expression in renal cancer cells (Jung et al., 2006). In addition, curcumin treatment of Burkitt’s lymphoma cell lines caused upregulation of DR-5 (Hussain et al., 2008). 

In another study on human breast cancer cells, curcumin-induced production of ROS did not affect total expression of DR-5, but it enhanced mobilization of DR-5 to the plasma membrane (Park et al., 2013). 

Overexpression of DR-4 or DR-5 can induce apoptosis independent of ligand binding in vitro, suggesting that the initiation of apoptosis can bypass ligand binding. The signal can be directly transduced to downstream sites that activate caspases(Schulze-Osthoff et al., 1998). Several studies have also reported that DRs, such as tumor necrosis factor receptor (TNF-R), can induce apoptosis in a ligand-independent manner(Fumarola et al., 2001). We; therefore, raised the possibility that curcumin might induce apoptosis through a DR-5-mediated extrinsic pathway. Several studies have shown the role of curcumin in DR-5 up-regulation in cancer cells. This increasement leads to apoptosis induction in these cells (Jung et al., 2005; Jung et al., 2006). However, the dependency curcumin on DRs and apoptosismerits further investigation.

In conclusion, the results of this research demonstrated that curcumin suppressed cell proliferation and induced apoptosis in primary and metastatic tumor cells, but metastatic tumor cells were more resistant to apoptotic effects of curcumin. DR-5 was associated with apoptosis resistance. Up-regulation of DR-5 in response to curcumin treatment was significantly higher in primary tumor cells compared with metastatic cells. These findings provided important insights regarding the molecular mechanism of apoptosis resistance of metastatic tumor cells.

## Funding Statement

This work was supported by a grant from Tehran University of Medical Sciences (TUMS) Grant No 95-03-87-33016.

## Conflict of Interest

The author declares that they have no competing interests.
